# Reduced AMPK activation and increased HCAR activation drive anti-inflammatory response and neuroprotection in glaucoma

**DOI:** 10.1186/s12974-018-1346-7

**Published:** 2018-11-13

**Authors:** Mohammad Harun-Or-Rashid, Denise M. Inman

**Affiliations:** Department of Pharmaceutical Sciences, Northeast Ohio Medical University, 4209 State Route 44, Rootstown, OH 44272 USA

**Keywords:** Glaucoma, AMP-activated protein kinase, Ketogenic diet, Inflammation hydroxycarboxylic acid receptor

## Abstract

**Background:**

Glaucoma is a chronic degenerative disease for which inflammation is considered to play a pivotal role in the pathogenesis and progression. In this study, we examined the impact of a ketogenic diet on the inflammation evident in glaucoma as a follow-up to a recent set of experiments in which we determined that a ketogenic diet protected retinal ganglion cell structure and function.

**Methods:**

Both sexes of DBA/2J (D2) mice were placed on a ketogenic diet (keto) or standard rodent chow (untreated) for 8 weeks beginning at 9 months of age. DBA/2J-*Gpnmb*^*+*^ (D2G) mice were also used as a non-pathological genetic control for the D2 mice. Retina and optic nerve (ON) tissues were micro-dissected and used for the analysis of microglia activation, expression of pro- and anti-inflammatory molecules, and lactate- or ketone-mediated anti-inflammatory signaling. Data were analyzed by immunohistochemistry, quantitative RT-PCR, ELISA, western blot, and capillary tube-based electrophoresis techniques.

**Results:**

Microglia activation was observed in D2 retina and ON as documented by intense microglial-specific Iba1 immunolabeling of rounded-up and enlarged microglia. Ketogenic diet treatment reduced Iba1 expression and the activated microglial phenotype. We detected low energy-induced AMP-activated protein kinase (AMPK) phosphorylation in D2 retina and ON that triggered NF-κB p65 signaling through its nuclear translocation. NF-κB induced pro-inflammatory TNF-α, IL-6, and NOS2 expression in D2 retina and ON. However, treatment with the ketogenic diet reduced AMPK phosphorylation, NF-κB p65 nuclear translocation, and expression of pro-inflammatory molecules. The ketogenic diet also induced expression of anti-inflammatory agents Il-4 and Arginase-1 in D2 retina and ON. Increased expression of hydroxycarboxylic acid receptor 1 (HCAR1) after ketogenic diet treatment was observed. HCAR1 stimulation by lactate or ketones from the ketogenic diet reduced inflammasome formation, as shown by reduced mRNA and protein expression of NLRP3 and IL-1β. We also detected increased levels of Arrestin β-2 protein, an adapter protein required for HCAR1 signaling.

**Conclusion:**

Our data demonstrate that the AMPK activation apparent in the glaucomatous retina and ON triggers NF-κB signaling and consequently induces a pro-inflammatory response. The ketogenic diet resolves energy demand and ameliorates the inflammation by inhibition of AMPK activation and stimulation of HCAR1-ARRB2 signaling that inhibits NLRP3 inflammasome-mediated inflammation. Thus, these findings depict a neuroprotective mechanism of the ketogenic diet in controlling inflammation and suggest potential therapeutic targets for inflammatory neurodegenerative diseases, including glaucoma.

## Background

Glaucoma is a chronic optic neuropathy that progressively damages the optic nerve (ON) and leads to retinal ganglion cell (RGC) loss [1, 2]. It is one of the leading causes of irreversible vision loss worldwide [3]. A critical risk factor for ON damage is elevated intraocular pressure (IOP), but how RGC dysfunction and degeneration occurs is not fully understood [4, 5].

Energy compromise can contribute to axon loss through axon degeneration initiated by nicotinamide mononucleotide adenylyltransferase-2 (NMNAT2) depletion [6] exacerbated by loss of membrane potential that initiates Ca^++^ dysregulation and cytoskeletal breakdown [7]. NMNAT2 loss could critically tie axon maintenance and metabolism with the optic neuropathy of glaucoma. Our recent studies in the DBA/2J (D2) model of glaucoma revealed that ONs of D2 mice are metabolically vulnerable and exhibit chronic metabolic stress [8]. The D2 ON exhibited lower ATP levels, low substrate availability, transporter downregulation, and mitochondrial defects [8–10]. Axonal metabolic decline can be reversed through increased substrate availability and upregulation of monocarboxylic transporters as a result of placing mice on a ketogenic diet [8]. A ketogenic diet is primarily composed of fat with a moderate level of protein and little to no carbohydrate. Ketogenic diet induces hepatic ketogenic metabolism and produces ketone bodies, primarily β-hydroxybutyrate (βHB) [11]. Ketogenic diet has been used as a therapy for neurological disorders, including in patients with Alzheimer’s disease, and in animal models of multiple sclerosis, Parkinson’s disease, and amyotrophic lateral sclerosis [12–16]. An ongoing clinical trial has used ketogenic diet to treat Alzheimer’s disease (study ID: NCT02912936), leading to reduction of short-term memory loss [17]. The exact mechanism of action of ketogenic diet in neuroprotection is not entirely known. Recent studies suggested ketogenic diet attenuates oxidative stress, inhibits class I histone deacetylases, promotes Nrf2 activation to upregulate antioxidants, and inhibits NF-κB to limit inflammation [18, 19]. In the treatment of epilepsy, the ketogenic diet inhibits neuronal activation through, among other changes, increased GABAergic output and lowered presynaptic excitatory neurotransmitter release [20].

AMP-activated protein kinase (AMPK) is a ubiquitously expressed Ser/Thr kinase that acts as an energy sensor by monitoring the AMP/ATP level and regulates cellular metabolism through ATP restoration [21]. In addition to acting as a key regulator of cellular energy dynamics, AMPK signaling is involved in regulating inflammation. Studies suggest AMPK signaling regulates NF-κB activation, enabling release of pro-inflammatory cytokines [22, 23]. NF-κB is a critical regulator of inflammatory response and immunity. There are five family members of NF-κB, including NF-κB p50, NF-κB p52, NF-κB p65, RELB, and c-REL that can form homo- and hetero-dimers [24]. The most conventional dimer form is the NF-κB p65-p50 heterodimer that remains bound to the IκB family of inhibitory proteins in the cytoplasm [25]. Phosphorylation of the NF-κB/IκB complex on IκB serine residues results in dissociation of IκB from NF-κB, leading to NF-κB p65 translocation to the nucleus. The translocated NF-κB p65 binds to DNA sequences in the promoter regions of specific genes such as tumor necrosis factor alpha (TNF-α), interleukin 1 beta (IL-1β), and interleukin 6 (IL-6), inducing their transcription [26].

Lactate or ketone bodies produced from the ketogenic diet can bind and activate hydroxycarboxylic acid receptors (HCARs). Three receptor subtypes have been identified: HCAR1 (also known as GPR81), HCAR2 (GPR109A), and HCAR3 (GPR109B). The HCAR3 subtype is evolutionary lost in rodents [27]. The HCARs are G-protein-coupled receptors (GPCRs) associated with downregulation of cyclic adenosine monophosphate (cAMP) through G_i_ signaling that inhibits lipolysis in adipocytes [28]. HCAR expression is not confined to adipocytes but is also present on neutrophils, on tissue macrophages, in the brain on neurons and astrocytes of the hippocampus and the cerebellum, and in the retina, including on Müller glia [27, 29, 30]. HCAR activation by lactate or ketone bodies has a direct anti-inflammatory effect. Lactate activation of HCAR1 reduces liver and pancreatic injuries via inhibition of inflammasome-mediated inflammation [31]. Stimulation of HCAR2 by βHB in a mouse model of stroke induces neuroprotection via the activity of bone marrow-derived macrophages that infiltrate the brain [32]. Studies suggested the anti-inflammatory effect of lactate is solely dependent on HCAR1 and its adapter protein Arrestin *β*-2 (ARRB2), which ultimately reduces the activation of the NLRP3 inflammasome [32]. Inhibition of NLRP3 inflammasome activation prevents proteolytic cleavage and activation of the pro-inflammatory cytokine IL-1β, the gatekeeper cytokine that regulates most of the inflammatory response [33].

Low energy can drive inflammation [22]. Compromised energy has been observed in glaucoma [8]; additionally, inflammation has been implicated in the mechanism of glaucomatous cell dysfunction and death [34]. In this study, we investigated whether the metabolic vulnerability observed in glaucoma contributes to an extended inflammatory response, then tested the hypothesis that the ketogenic diet resolves the inflammatory response in glaucoma. Our data indicate that low energy drives inflammation in D2 chronic glaucoma mice as shown by phosphorylation of AMPK that leads to activation of NF-κB and cytokine production and release. Treatment with the ketogenic diet ameliorates inflammation through inhibition of AMPK activation and HCAR1-mediated inhibition of the NLRP3 inflammasome.

## Methods

### Animals

Both sexes of DBA/2J (D2) and DBA/2J-*Gpnmb* + (D2G) mice were purchased from The Jackson Laboratory (Bar Harbor, ME, USA) and housed at Northeast Ohio Medical University. The D2 mouse has mutations in the *Tyrp1* and *Gpnmb* genes that cause iris stromal atrophy and iris pigment dispersion disease, leading to age-related elevation of intraocular pressure and ocular hypertension-related retinal ganglion cell death [35]. The D2G mouse shares the D2 genetic background but carries a wildtype allele of the *Gpnmb* gene and does not develop pigmentary glaucoma. These experiments used 34 D2 mice split across two treatment groups and 6 D2G mice (Table 1). All animal procedures were approved by the Institutional Animal Care and Use Committee and performed in accordance with the ARVO Statement for the Use of Animals in Ophthalmic and Vision Research.

**Table 1:** Ketogenic and control mouse indices

Mice		Baseline IOP (mmHg)	Terminal IOP (mmHg)	Baseline weight (g)	Terminal weight (g)	Food intake by week (g)	kCal by week	Plasma βHB levels (mM)
Keto D2	Male (*n* = 10)	17.2 ± 1.3	18.5 ± 1.3	33.2 ± 1.7	40.2 ± 2.1	18.5 ± 0.7	116.5 ± 0.8	0.53 ± 0.042
	Female (*n* = 8)	16.5 ± 0.8	17.4 ± 1.2	26.6 ± 0.8	29.5 ± 1.5	12.5 ± 0.4	78.7 ± 1.5	0.40 ± 0.03
Control D2	Male (*n* = 10)	17.5 ± 0.2	18.1 ± 0.8	32.2 ± 1.4	32.0 ± 1.2	30.5 ± 1.2	119.8 ± 1.1	0.102 ± 0.01
	Female (*n* = 6)	16.4 ± 1.0	17.5 ± 1.0	26.5 ± 0.9	26.1 ± 1.2	24.5 ± 1.6	95.5 ± 1.2	0.090 ± 0.04
D2G	Male (*n* = 3)	12.5 ± 0.1	12.2 ± 0.1	30.5 ± 1.5	31.0 ± 1.5	29.5 ± 1.5	115.5 ± 1.4	0.07 ± 0.01
	Female (*n* = 3)	12.1 ± 0.2	11.8 ± 0.2	25.5 ± 1.0	26.2 ± 1.1	22.1 ± 1.2	86.2 ± 1.3	0.06 ± 0.02

All values ± SEM

*n* number of mice

### Intraocular pressure measurement

The Tono-Lab rebound tonometer (Tiolat-Oy, Finland) calibrated for mice was used to measure intraocular pressure (IOP). Mice were anesthetized (2.5% isoflurane delivered by vaporizer with oxygen) prior to IOP measurement and 10–15 measures were taken, and values were averaged. Both baseline and terminal IOP were measured for untreated and keto D2 and D2G mice (Table 1). All IOP measurements were carried out within 3 min of anesthetization in order to avoid any anesthetic-induced reduction of IOP [36].

### Diets

Both D2 and D2G mice were fed by standard lab chow (Formulab Diet 5008; 26.8% protein, 56.4% carbohydrate, 16.7% fat) ad libitum. At 9 months of age, D2 mice were switched from standard lab chow to a very low carbohydrate, ketogenic diet (D12369B, Research Diets) for 8 weeks. The composition of the ketogenic diet was 10.4% protein, 0.1% carbohydrate, and 89.5% fat. The ketogenic diet was a soft dough, given to the mice in small stainless-steel bowl. A nylon chew bar (I-Chews from Animal Specialties & Provisions) was placed in the cage in order to provide a non-nutritive chewing surface to compensate for potential tooth overgrowth anticipated in mice provided the ketogenic diet. All mice and their food intake were weighed once weekly over 8 weeks (Table 1). Ketone levels were measured from tail vein blood using a Nova Max Plus hand-held ketone testing device once weekly from a random sample of untreated and ketogenic diet (keto) D2 mice. Final measurements of serum βHB levels were measured using a βHB assay kit (700190, Cayman Chemical, Ann Arbor, MI, USA) from blood collected at euthanasia (Table 1).

### Immunohistochemistry

Freshly enucleated eyes were immersion fixed in 4% paraformaldehyde for 1 h then cryoprotected in 30% sucrose with 0.02% sodium azide. Lenses were removed from the globes that were then embedded in OCT (Sakura Finetek, Torrance, CA, USA) and frozen on dry ice. Optic nerves were fine-dissected from the brain and similarly cryoprotected and embedded in OCT. Both eyes and optic nerves were sectioned at 10 μm on a cryostat. For immunohistochemistry, all tissues were washed in 0.1 M PBS, then incubated in blocking solution (5% donkey serum, 0.5% Triton X-100 in 0.1 M PBS) for 1 h, incubated with primary antibody (diluted in 0.5% BSA, 0.9% NaCl, 0.5% Triton X-100 in 0.1 M PBS) for 24 h at 4 °C, then washed and incubated with secondary antibody for 2 h at room temperature, washed, then cover slipped with DAPI Fluoromount-G (Southern Biotech). Primary antibodies were rabbit anti-Iba1 (1:300, 019-19741, WAKO, Richmond, VA, USA), mouse anti-TNF-α (1:50, ab1793, Abcam, Cambridge, MA, USA), rabbit anti-NF-κB p65 (1:100, SAB4502610, Sigma-Aldrich, St. Louis, MO, USA), mouse anti-NOS2 (1:50, sc-7271, Santa Cruz Biotechnology, Dallas, TX, USA), mouse anti-Arginase 1 (1:50, sc-271430, Santa Cruz), mouse anti-HCAR1 (1:200, SAB1300090, Sigma-Aldrich), and goat anti-GFAP (1:250, ab53554, Abcam). Secondary antibodies were obtained from Jackson ImmunoResearch, used at 1:250 dilution, and raised in donkey against the species appropriate to the primary antibody: anti-mouse Alexa Fluor 488 (715-545-150), anti-mouse Alexa Fluor 594 (711-585-150), anti-goat Alexa Fluor 488 (705545-147), anti-rabbit Alexa Fluor 647 (711-605-152), and anti-rabbit Alexa Fluor 594 (711-545-152).

### Microscopy

A Leica DMi8 confocal microscope integrated with Leica application Suite X 3.1.1.15751 (Leica Microsystems, Buffalo Grove, IL, USA) was used for microscopy. The same exposure time settings were used for capturing all the photomicrographs among all groups. Photomicrographs were obtained from the central part of retina and from proximal region of optic nerves. Immunoreactivity was quantified by using an optical density measurement within regions of interest (ROIs) using Fiji-ImageJ [37]. Minimum eight ROIs were used per antigen for quantification. For microglial quantification, five sections per retina and ON and five retinas and ONs for each group (a total of 25 sections per tissue) were used. Microglia were only counted if the DAPI-stained nucleus could be identified.

### RNA analysis

Retina was dissected from freshly enucleated eyes, and total RNA was isolated using the Trizol extraction kit (Thermo-Fisher Scientific, Waltham, MA, USA). cDNA was synthesized from 1 μg DNase-treated RNA by using cDNA synthesis kit (Verso cDNA Synthesis Kit, Thermo-Fisher Scientific) then analyzed using real-time quantitative PCR with a QuantStudio™ 6 Flex Real-Time PCR System instrument (Applied Biosystems). Both TaqMan and SYBR Green assays were used for mRNA analysis. TaqMan Gene Expression Assays from Thermo-Fisher Scientific with a FAM reporter dye at the 5′ end of the TaqMan MGB probe were used; TaqMan assay probes were *Hcar1* (Mm00558586_s1), *Hcar2* (Mm01199527_s1), and *Hprt* (Mm00446968_m1). SYBR Green assays were performed using previously published primers [38] including *Il-6* For-TCCATCCAGTTGCCT TCTTG and Rev-ATTGCCATTGCACAACTCTTTT, *Nos2* For-TCACGCTTGGGTCTTGTT and Rev-CAGGTCACTTTGGTAGGATTT, *Il-4* For ACCACAGAGAGTGAGCTCGT and Rev-AGGCATCGAAAAGCCCGAAA, *Arg1* For-GGACCTGGCCTTTGTTGATG and Rev-AGACCGTGGGTTCTTCACAATT, *Nlrp3* For-TGCTCTTCACTGCTATCAAGCCCT and Rev-ACAAGCCTTTGCTCCAGACCCTAT, *Il-1β* For-TGGACCTTCCAGGATGAGGACA and Rev-GTTCATCTCGGAGCCTGTAGTG, and *Hprt* For-CAGTCAACGGGGGACATAAA and Rev-AGAGGTCCTTTTCACCAGCAA. All primers were obtained from Thermo-Fisher Scientific. *Hprt* was used as the housekeeping gene, chosen after a comparison of *Actb*, *Rpl*, *Hprt*, and *GlucB*, which showed *Hprt* had the most stable gene expression across age and strain using retina and optic nerve mRNA.

### Protein analysis

**Fig. 1:**
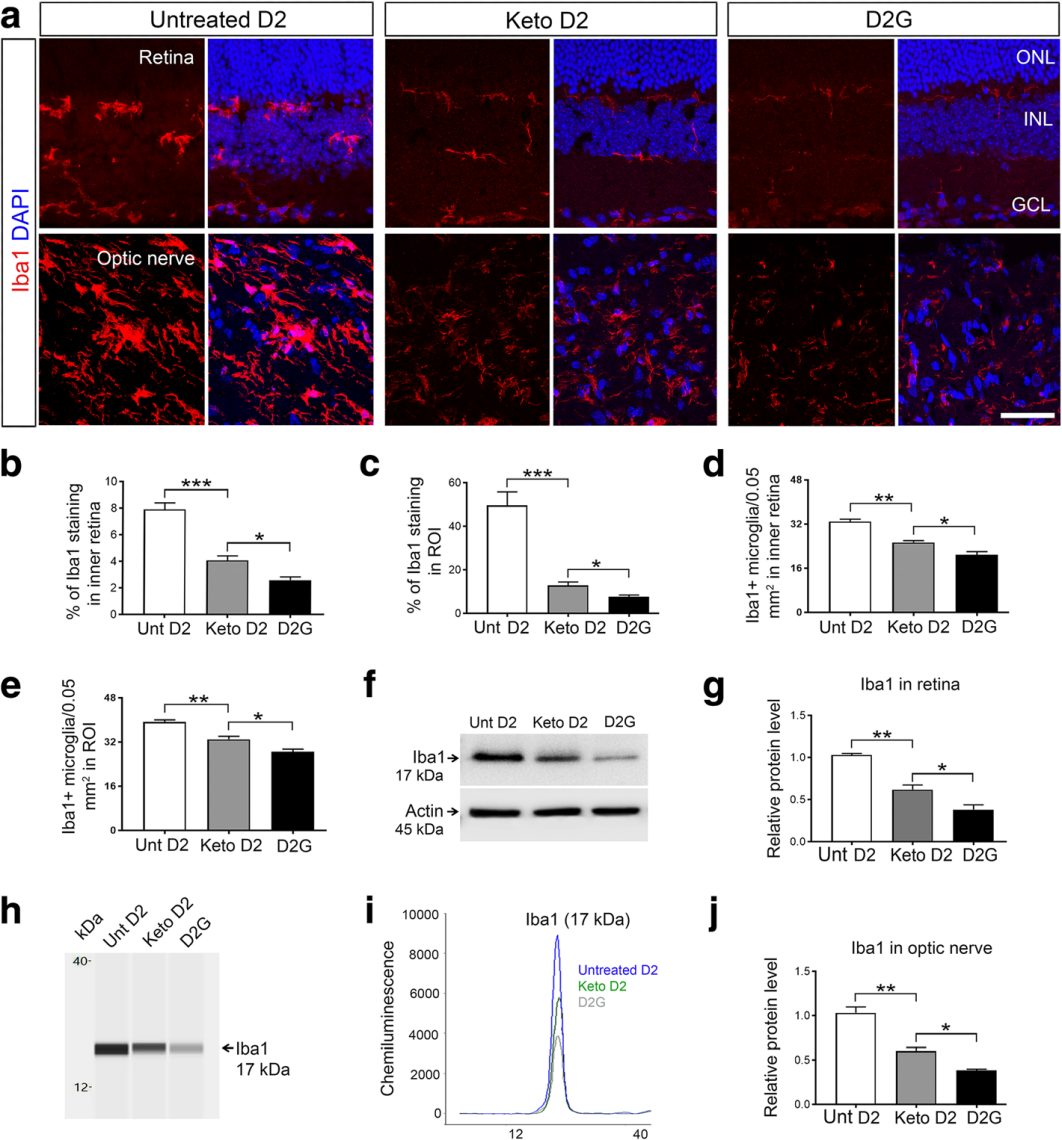
Iba1 expression in the retina and ON of D2 and D2G mice. **a** Immunohistochemical analysis of Iba1 (red) expression in untreated (Unt) and keto D2 and D2G retinas and ONs. DAPI (blue) stains nuclei. **b**, **c** Percentage of mean fluorescence of Iba1 in the inner retina (**b**), *p = 0.0369, ***p = 0.0001, and in the region of interest (ROI) of the proximal ON (**c**), *p = 0.0415, ***p = 0.0001. **d**, **e** Quantification of Iba1+ microglia in the inner retina (**d**), *p = 0.0341, ***p = 0.0001, and in the ROI of the proximal ON (**e**), *p = 0.0245, ***p = 0.0001. For **b**–**e**, five retinas and ON from each group were analyzed, with five sections from each individual retina or ON. **f** Western blot analysis of Iba1 protein expression in retinas. **g** Bar graph with densitometry of Iba1 levels normalized to actin (β isoform) levels, *p = 0.0293, **p = 0.0022; *n* = 3 blots per group, each with independent samples. **h**–**j** Capillary tube electrophoresis of Iba1 protein in ON, normalized to actin levels. Typical capillary electrophoresis output, showing digital view of bands within capillary tube (**h**), digital graphical plot of HRP signal produced by HRP-conjugated secondary antibody that is bound to the antigen of interest (**i**); bar graph represents the areas of HRP signal normalized to areas of actin HRP signal (**j**), *p = 0.0310, ***p = 0.0013; *n* = 3 biological replicates per group. All bar graphs are the mean ± SEM, *n* = 5, analyzed by two-way, unpaired *t* test. Scale bar, 20 μm

Fresh retina and optic nerve tissues from D2 and D2G retina and ON were isolated and flash frozen in liquid nitrogen until homogenization in T-PER buffer (Thermo-Fisher Scientific) with HALT protease and phosphatase inhibitors (78442, Thermo-Fisher Scientific) to prevent enzymatic degradation of protein. A Branson Sonicator using three 3-s pulses at 10% amplitude was used for sonicating tissues. All protein samples were spun down at 10,000*g* for 10 min; supernatants were collected, and the nuclear protein fraction was extracted using the EpiQuik total histone extraction kit (# OP-0006, EpiGentek, Farmingdale, NY, USA). Both cell lysate and nuclear protein fractions were analyzed by western blot (WB) and capillary tube-based electrophoresis immunoassay using the Wes, a ProteinSimple instrument (San Jose, CA, USA) that separates proteins by electrical charge in capillary tubes and allows binding of antibodies and detection within the capillary. The Wes is suitable for analysis of very small amounts of protein, allowing us to assay individual optic nerve and quantify specific proteins within a lysate. All the Wes results (see Fig. 1f–h) were normalized to β-actin protein level. Primary antibodies used were rabbit anti-Iba1 (1:1000 for WB, 1:50 for Wes; 019-19741, WAKO), mouse anti-AMPK α1 2B7 (1:200 for Wes, NBP2-2217, Novus Biological, Littleton, CO, USA), anti-mouse AMPK α1_Thr 172_ (1:50 for Wes, NBP1-74502, Novus Biological), mouse anti-TNF-α (1:1000 for WB, 1:50 for Wes; ab1793, Abcam), mouse anti-NF-κB p65 (1:1000 for WB, 1:25 for Wes; 6956, Cell signaling, Danvers, MA, USA), mouse anti-Histone H3 (1:1000 for WB, 1:25 for Wes; 61473, Active Motif Carlsbad, CA, USA), mouse anti-Actin (β isoform) (1:1000 for WB, 1:25 for Wes; NB600-501, Novus Biological), mouse anti-Arginase 1 (1:100 for WB, 1:25 for Wes; sc-271430, Santa Cruz), mouse anti-HCAR1 (1:1000 for WB, 1:25 for Wes; SAB1300090, Sigma-Aldrich), mouse anti-NLRP3 (1:1000 for WB, AG-20B-0014-C100, AdipoGen, San Diego, CA, USA), rabbit anti-IL-1β (1:1000, AB1413-I, Millipore Sigma, Burlington, MA, USA), and mouse anti-ARRB2 (1:100 for WB, 1:25 for Wes; sc-365445, Santa Cruz). Secondary antibodies used were donkey anti-rabbit IgG, HRP conjugated (1:5000, 711-035-152, Jackson ImmunoResearch), and goat anti-mouse IgG, HRP conjugated (1:5000, sc-2031, Santa Cruz Biotechnology).

### Enzyme-linked immunosorbent assay (ELISA)

Optic nerve protein samples were extracted as described above, and ELISA was performed using the mouse cytokine ELISA plate array I (EA-4005, Signosis, Santa Clara, CA, USA). ON protein lysate (100 μL per well) was added to the plate and incubated 24 h at 4 °C with gentle shaking. Two wells were designated blanks by adding diluent buffer instead of protein sample. After incubation, each well was aspirated and washed with washing buffer and then incubated with 100 μl biotin-labeled antibody mixture for 2 h at room temperature, then aspirated, washed, and incubated with 100 μl streptavidin-HRP conjugate for 1 h at room temperature with gentle shaking. After aspiration and wash, the plate was incubated with 100 μl substrate for 45 min at room temperature. The substrate reaction was stopped by adding 50 μl stop solution that changed color from blue to yellow, and then optical density was determined with a microtiter plate reader at 450 nm. Output of ELISA was normalized by total protein level as quantified by the Pierce™ BCA Protein Assay Kit (23225, Thermo-Fisher Scientific).

### Statistical analysis

All statistical analyses were performed by GraphPad Prism Version 7.02 (GraphPad software, Inc., La Jolla, CA, 92037 USA). Data were analyzed by unpaired, two-tailed *t* test when comparing two groups. One-way ANOVA and Tukey’s multiple comparison post hoc test was used when comparing across multiple groups. All data were presented as the mean ± SEM, and *p* < 0.05 was considered significant difference. In the text and figure legends, “*n*” represents number of samples, either retina or ON.

## Results

At 9 months of age, D2 mice have significant IOP elevation (D2 average IOP 18.1 ± 1.3 mmHg versus D2G average IOP 11.9 ± 0.2 mmHg, as shown in Table 1), but have not yet undergone significant optic nerve degeneration and retinal ganglion cell loss [1, 8]. The D2 mice were placed on the ketogenic diet at 9 months of age to probe the role of inflammation in the neuroprotection observed with ketogenic diet treatment [8].

### Ketogenic diet reduces microglia number

Microglia monitor the microenvironment in the central nervous system (CNS) and display small somata and ramified morphology with many complex processes in their resting stage. During injury or any kind of CNS pathology, microglia become activated and change their morphology [39]. We used the microglia-specific marker Iba1 [40] to monitor the activation of microglia in the retina and optic nerve (ON) of D2 glaucoma mice and investigated whether feeding D2 glaucoma mice a ketogenic diet affected microglia activation or number. Using immunohistochemistry, we detected Iba1-positive (+) microglia in the retina and ON tissues. We observed highly intense Iba1+ microglia in untreated D2 retina and ONs, whereas in the ketogenic diet-fed D2 retina and ON, Iba1 staining intensity was reduced (Fig. 1a–c). Quantification of Iba1+ microglia showed significantly increased numbers of Iba1+ microglia in untreated D2 retina and ON compared to keto D2 retina and ON (Fig. 1d, e), though microglial numbers in keto D2 retina and ON were also significantly higher than age-matched D2G tissue. We quantified Iba1 protein level in the retina and ON by western blot analysis and capillary tube electrophoresis respectively, detecting significantly decreased Iba1 protein in keto D2 retina and ON compared to untreated D2 retina (Fig. 1f, g) and ON (Fig. 1h–j). Thus, these results indicate that the ketogenic diet limits microglia activation in D2 retina and ON.

### Ketogenic diet regulates AMPK activation, TNF-α release, and nuclear translocation of NF-κB p65

**Fig. 2:**
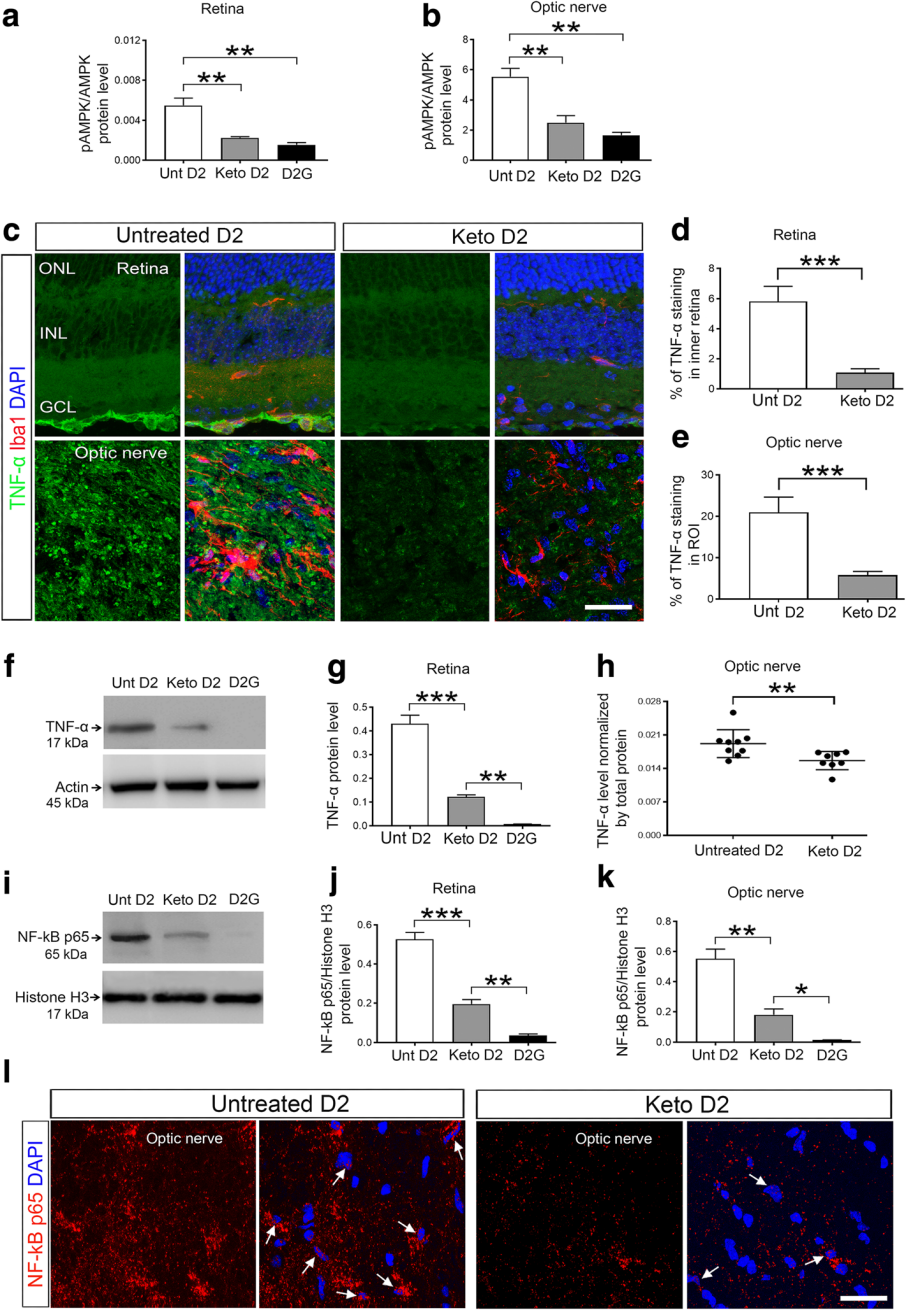
Analysis of AMPK phosphorylation, TNF-α expression, and nuclear translocation of NF-κB p65 in D2 and D2G retina and ON. **a**, **b** The ratio of pAMPK to AMPK protein in the retina (**a**), **p = 0.0034, **p = 0.0016; *n* = 4 per group and ON (**b**), **p = 0.0018, **p = 0.0013, as analyzed by capillary electrophoresis; *n* = 6 Keto D2 and 3 D2G ON per group. **c** Immunohistochemical analysis of TNF-α (green) expression in untreated (Unt) and keto D2 retinas and ONs. Iba1 (red) labels microglia (**c**) and DAPI (blue) stains nuclei. **d**, **e** Percentage of mean fluorescence of TNF-α in the inner retina (**d**), ***p = 0.0001, and in the ROI of the proximal ON (**e**), ***p = 0.0001; *n* = 5 retinae per group, with five sections analyzed per retina. **f** Western blot analysis of TNF-α in untreated and keto D2 and D2G retinas. **g** Bar graph showing the quantification by densitometry of TNF-α protein levels normalized to actin levels in the retina, **p = 0.0035, ***p = 0.0001; *n* = 3 blots per group, each with independent samples. **h** TNF-α protein levels in ON analyzed by ELISA, **p = 0.0032; *n* = 8 per group. **i** Western blot analysis of NF-κB p65 levels in retinal nuclear protein fraction. **j** Quantification by densitometry of NF-κB p65 levels normalized to total histone H3 levels, **p = 0.0032, ***p = 0.0001; *n* = 3 blots per group, each with independent samples. **k** NF-κB p65 levels in nuclear fraction of the ON protein normalized to total histone H3 levels, as quantified with capillary electrophoresis, *p = 0.00593, **p = 0.0023; *n* = 3 ON per group. **l** NF-κB p65 (red) immunohistochemistry in the ON. Arrows indicate nuclear translocation of NF-κB. All bar graphs are presented as the mean ± SEM, *n* = 5–9 analyzed by two-tailed unpaired *t* test. **d**, **l** Scale bar, 20 μm

AMPK is a major energy sensor and regulator that promotes ATP production and inhibits ATP consumption during energy shortage [41]. In addition to its key role in energy balance, it can stimulate a variety of transcription factors and signal transduction molecules, including inflammatory cytokine release and activation of NF-κB signaling [22, 23]. We examined whether the ketogenic diet impacted AMPK signaling, cytokine release, and NF-κB activation in D2 mice. We detected significantly lower protein levels of pAMPK from the retina (Fig. 2a) and ON (Fig. 2b) from D2 mice on the ketogenic diet as compared to D2 mice on the control diet. We measured expression of the cytokine TNF-α in the retina and ON of D2 mice, revealing increased levels of TNF-α staining in D2 control diet retina (Fig. 2c, d) and ON (Fig. 2c, e) that were significantly reduced by the ketogenic diet (Fig. 2c–e). TNF-α levels in D2 retina quantified by western blot and in the ON by enzyme-linked immunosorbent assay (ELISA) showed increased levels of TNF-α in both D2 control diet retina (Fig. 2f, g) and ON (Fig. 2h) that were significantly reduced in D2 mice on the ketogenic diet (Fig. 2f–h).

TNF-α and interleukin 1-beta (IL-1β) can induce NF-κB p65, leading to its nuclear translocation and promotion of many inflammatory genes [42]. We extracted nuclear protein fractions from the retina and ON then measured NF-κB p65 levels, detecting elevated levels of nuclear NF-κB p65 in control diet D2 retina and ON that were significantly decreased in keto D2 retina (Fig. 2i, j) and ON (Fig. 2k), but still significantly higher than levels observed in the D2G retina and ON (Fig. 2j, k). Immunohistochemistry analysis of ON sections showed increased NF-κB p65 immunostaining and nuclear translocation in untreated D2 ON compared to keto D2 ON (Fig. 2l). Collectively, these results suggest that the ketogenic diet satisfies energy demand, thereby inhibiting AMPK activation and preventing AMPK-induced TNF-α release and subsequent NF-κB p65 transcriptional activity.

### Ketogenic diet reduces pro-inflammatory response and enhances anti-inflammatory signals

**Fig. 3:**
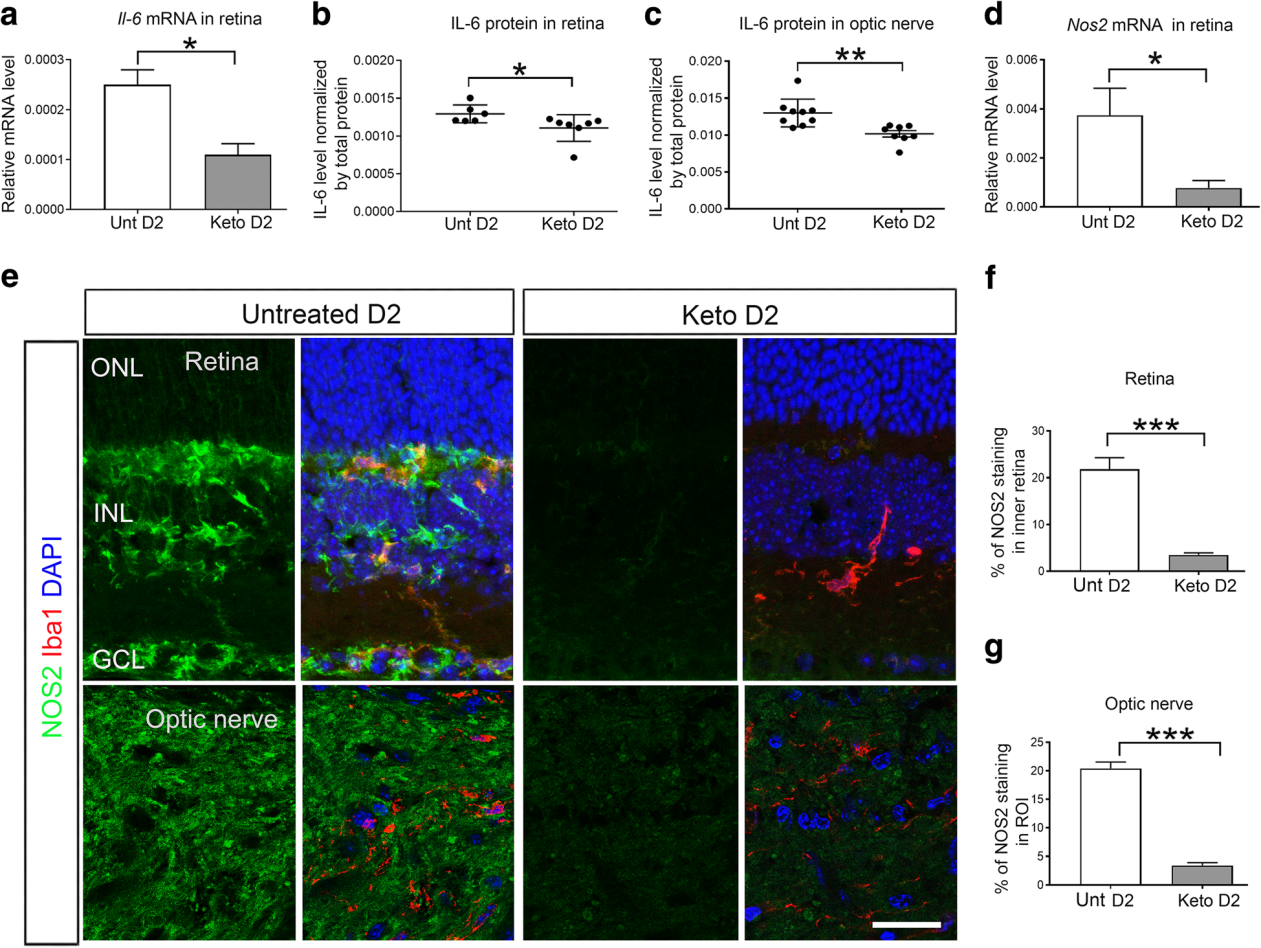
Ketogenic diet inhibits pro-inflammatory IL-6 and NOS2 expression in D2 retina and ON. **a** Bar graph showing qRT-PCR analysis of *Il-6* mRNA level in untreated (Unt) and keto D2 retinas normalized to *Hprt* mRNA level, *p = 0.0477; *n* = 8 samples per group. **b**, **c** IL-6 protein levels in the retina (**b**), *p = 0.049, and ON (**c**), **p = 0.0023, analyzed by ELISA; *n* = 8 samples per group. **d** *Nos2* mRNA levels in the retina normalized by *Hprt* mRNA levels, *p = 0.0426; *n* = 8 samples per group. **e** Immunohistochemical analysis of NOS2 (green) in untreated and keto D2 retina and ON. Iba1 (red) labels microglia and DAPI (blue) stains nuclei. **f**, **g** Percentage of mean fluorescence of NOS2 in the inner retina (**f**), ***p = 0.0001, and in the ROI of the proximal ON (**g**), ***p = 0.0001; five retinas and ON from each group were analyzed, with five sections from each individual retina and ON. All bar graphs are presented as the mean ± SEM, analyzed by two-tailed unpaired *t* test. Scale bar, 20 μm

To further understand the consequences of the ketogenic diet on inflammation, we examined the expression of pro-inflammatory cytokine IL-6 and NOS2 in D2 glaucoma mice with or without the ketogenic diet. Quantitative RT-PCR analysis showed increased *Il-6* and *Nos2* mRNA expression in D2 retina, both of which are reduced after ketogenic diet treatment (Fig. 3a, d). Elevated IL-6 protein levels were detected in untreated D2 mice compared to the keto D2, in both the retina (Fig. 3b) and ON (Fig. 3c) by ELISA. Immunohistochemistry analysis of NOS2 showed intense immunolabeling in untreated D2 retina (Fig. 3e, f) and ON (Fig. 3e, g), whereas NOS2 labeling was very low in keto D2 retina and ON (Fig. 3e–g).

**Fig. 4:**
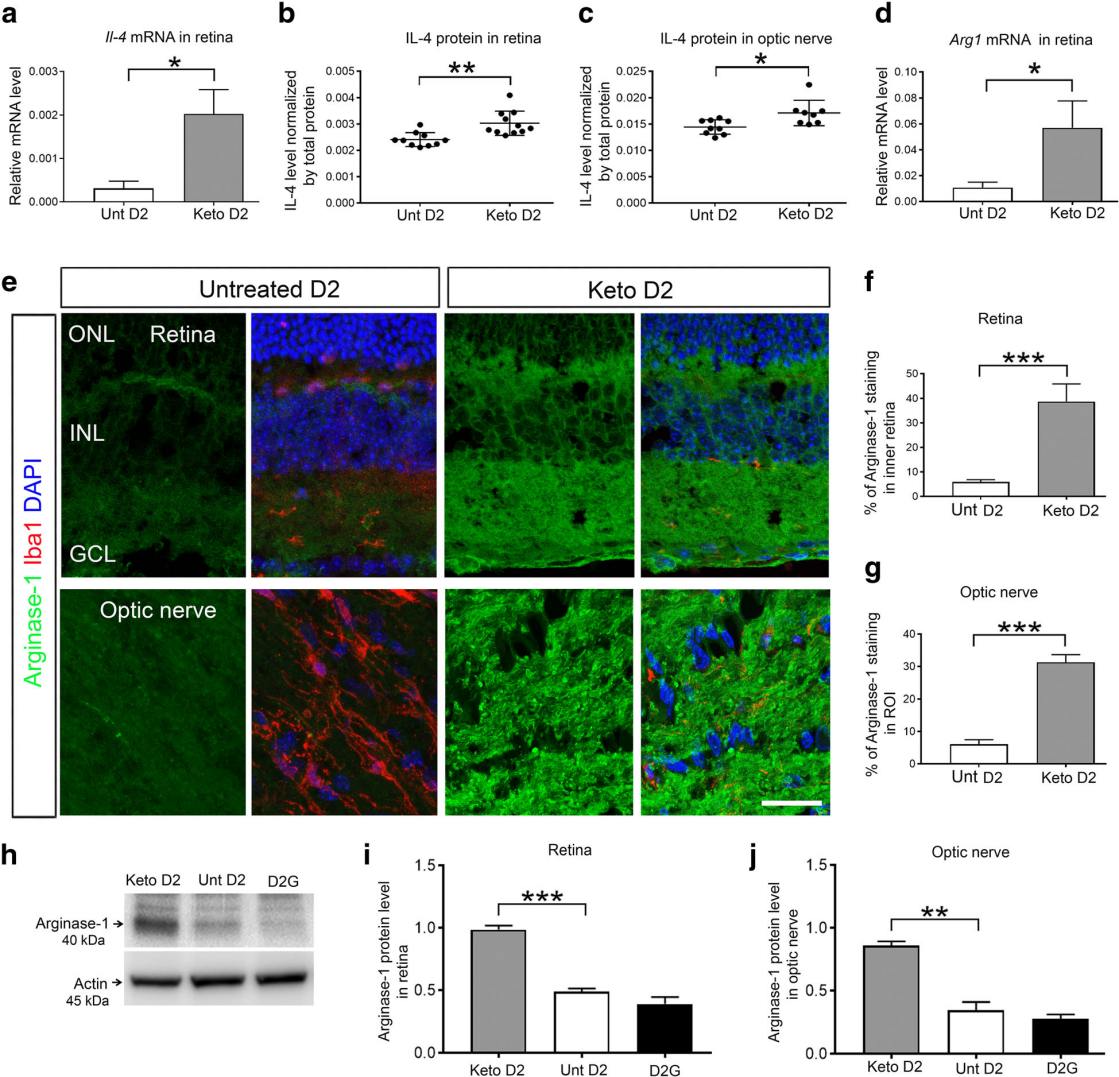
Ketogenic diet induces anti-inflammatory IL-4 and Arginase-1 expression in D2 retina and ON. **a** Bar graph showing *Il-4* mRNA normalized to *Hprt* mRNA by qRT-PCR in untreated (Unt) and keto D2 retina, *p = 0.0264; *n* = 8 samples per group. **b**, **c** IL-4 protein levels in the retina (**b**), **p = 0.0014, and ON (**c**), *p = 0.0121, analyzed by ELISA; *n* = 8 samples per group. **d** *Arg1* mRNA levels in retina normalized by *Hprt* mRNA levels, *p = 0.0368; *n* = 8 samples per group. **e** Immunohistochemical analysis of Arginase-1 (green) in the untreated and keto D2 retina and ON. Iba1 (red) labels microglia and DAPI stains nuclei. **f**, **g** Percentage of mean fluorescence of Arginase-1 in inner retina (**f**), ***p = 0.0001, and in the ROI of the proximal ON (**g**), ***p = 0.0001; five retinas and ON from each group were analyzed, with five sections from each individual retina and ON. **h** Western blot analysis of Arginase-1 protein in the retina. **i** Quantification by densitometry of Arginase-1 levels normalized to β-actin levels, ***p = 0.0001; *n* = 3 blots per group, each with independent samples. **j** Arginase-1 protein in the ON normalized to β-actin levels, as determined by capillary electrophoresis, **p = 0.0006; *n* = 3 ON per group. All bar graphs are presented as the mean ± SEM, analyzed by two-tailed unpaired *t* test. Scale bar, 20 μm

IL-4 and Arginase-1 are two cytokines released by immune cells exhibiting anti-inflammatory response [43]. We observed significantly higher levels of *Il-*4 and *Arg1* mRNA in keto D2 retina (Fig. 4a) and ON (Fig. 4d) compared to untreated D2 retina and ON. IL-4 protein levels were also significantly higher in keto D2 retina (Fig. 4b) and ON (Fig. 4c) compared to untreated D2. Immunohistochemistry analysis showed plentiful Arginase-1 labeling in keto retina (Fig. 4e, f) and ON (Fig. 4e, g), while barely detectable Arginase-1 labeling was observed in untreated D2 retina and ON (Fig. 4e–g). We quantified Arginase-1 protein levels in the retina and ON by western blot and capillary electrophoresis, respectively, detecting enhanced levels of Arginase-1 in keto D2 retina (Fig. 4h, i) and ON (Fig. 4j) compared to untreated D2 retina and ON. These results suggest that the ketogenic diet ameliorates neuroinflammation in D2 glaucoma mice by inhibiting pro-inflammatory while inducing anti-inflammatory response.

### Ketogenic diet induces HCAR1 expression

**Fig. 5:**
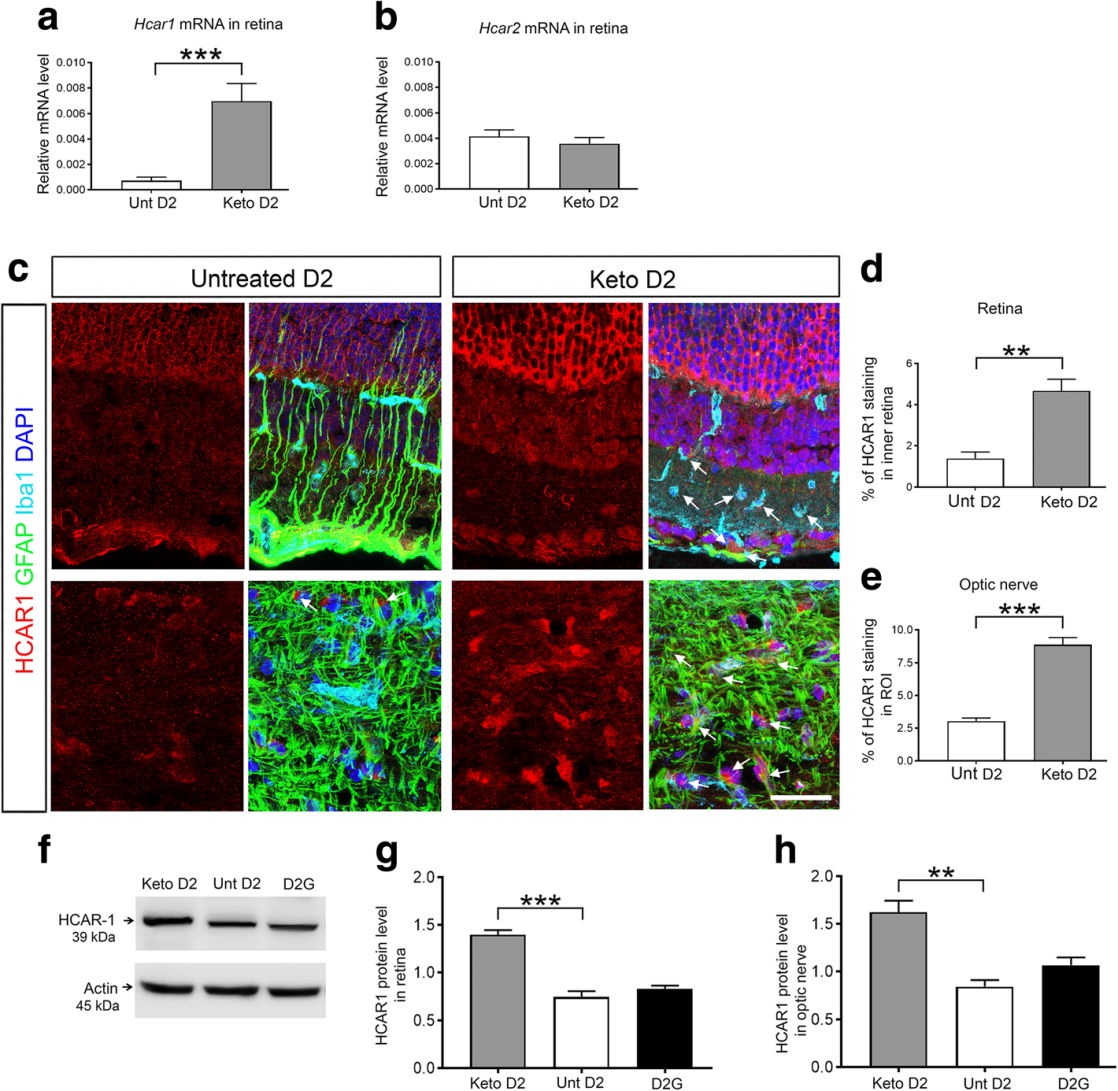
Analysis of HCAR expression in D2 and D2G retina and ON. **a**, **b** Bar graphs showing *Hcar1* (**a**), ***p = 0.0001, and *Hcar2* (**b**) *mRNA* normalized to *Hprt* mRNA by qRT-PCR; *n* = 8 ON per group. **c** Immunohistochemical analysis of HCAR1 (red) expression in the untreated (Unt) and keto D2 retina and ON. GFAP (green) labels Müller glia and astrocytes, Iba1 (cyan) labels microglia, and DAPI (blue) stains nuclei. Arrows indicate colocalization of HCAR1 with Iba1+ microglia and GFAP+ astrocytes. **d**, **e** Percentage of mean fluorescence of HCAR1 in the inner retina (**d**), **p = 0.0036, and in the ROI of the proximal ON (**e**), ***p = 0.0001; five retinae per group, with five sections/retina. **f** Western blot analysis of HCAR1 protein level in retina. **g** Quantification by densitometry of HCAR1 protein normalized to β-actin, ***p = 0.0008; *n* = 3 blots per group, each with independent samples. **h** HCAR1 protein in the ON normalized to β-actin levels, as determined by capillary electrophoresis, **p = 0.0025; *n* = 3 ON per group. All bar graphs are presented as the mean ± SEM, *n* = 5, analyzed by two-tailed unpaired *t* test. Scale bar, 20 μm

The primary ketone body produced by the liver on a ketogenic diet is β-hydroxybutyrate (βHB). Apart from use as an alternative energy source during energy demand, βHB can act as a signaling molecule via binding to hydroxyl-carboxylic acid receptors (HCARs) [11, 44]. As a result, we investigated whether the ketogenic diet alters the expression of HCARs in D2 glaucoma mice. By qRT-PCR analysis, we detected significantly increased levels of *Hcar1* mRNA in keto D2 retina compared to untreated D2 retina (Fig. 5a). We observed no change in *Hcar2* mRNA levels in D2 retina after ketogenic diet treatment (Fig. 5b). Immunohistochemistry analysis showed higher HCAR1 labeling in keto D2 retina (Fig. 5c, d) and ON (Fig. 5c, e) compared to untreated D2 retina and ON. HCAR1 colocalized with Iba1+ microglia and GFAP+ astrocyte somata (arrows), indicating the expression of HCAR1 in microglia and astrocytes. HCAR1 immunolabel was observed in many cell types in the retina, with high expression in the photoreceptor layer, the inner nuclear layer, and the ganglion cell layer. We quantified HCAR1 protein levels in retina and ON, detecting significantly higher levels of HCAR1 in both keto D2 retina (Fig. 5f, g) and ON (Fig. 5h) than in untreated D2 retina and ON. Thus, these data indicate that the ketogenic diet induces HCAR1 expression both in the retina and ON of D2 glaucoma mice.

### Ketogenic diet inhibits inflammasome component NLRP3 and inflammatory cytokine IL-1β release via activation of HCAR1-ARRB2 signaling

**Fig. 6:**
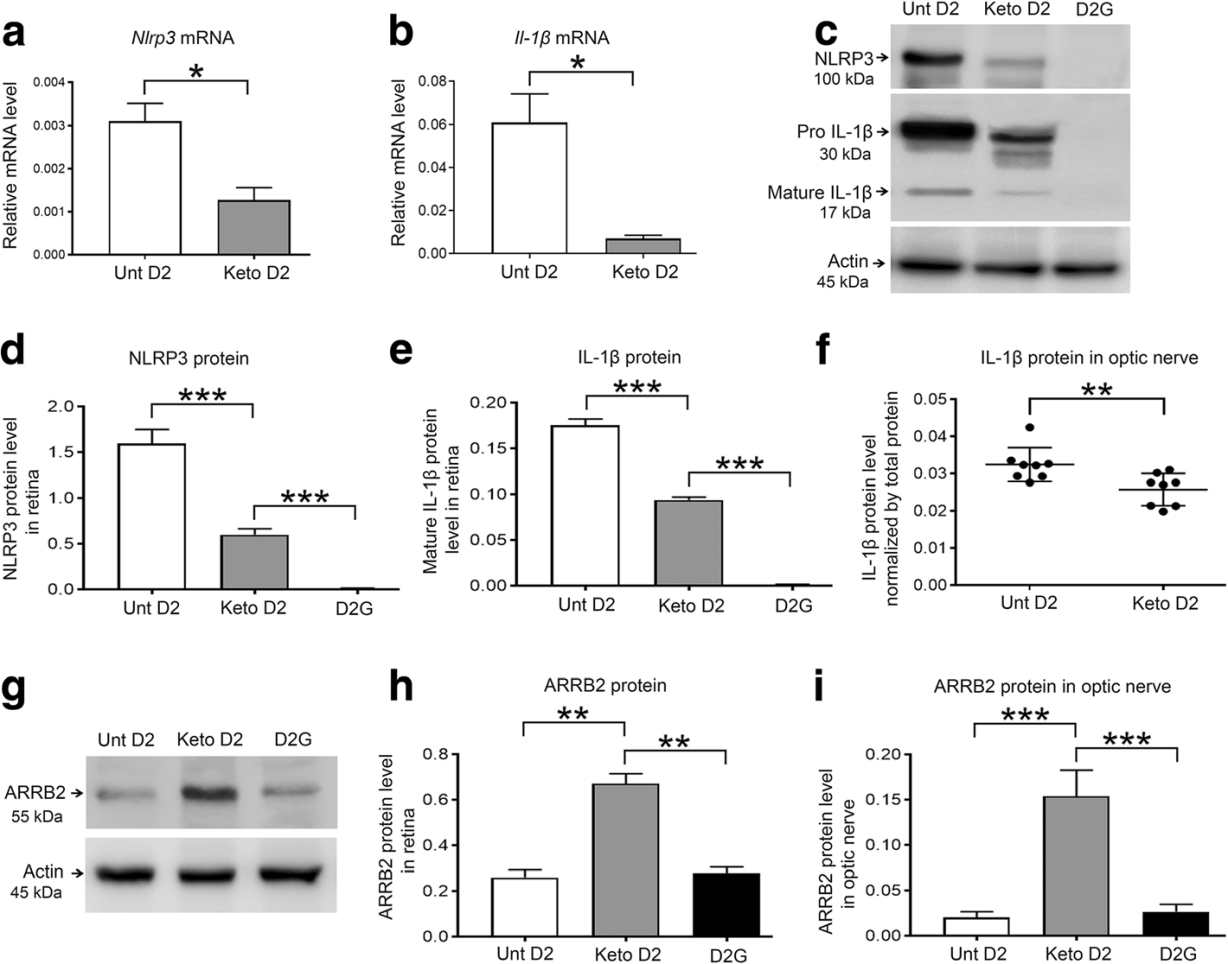
Analysis of NLRP3, IL-1β, and ARRB2 expression in untreated (Unt) and Keto D2 and D2G retina and ON. **a**, **b** *Nlrp3* (**a**), *p = 0.0219, and *Il-1β* (**b**), *p = 0.0151 *mRNA* levels normalized to *Hprt* mRNA levels determined by qRT-PCR; *n* = 8 samples per group. **c** Western blot analysis of NLRP3 and IL-1β protein levels in the retina. **d**, **e** Quantification by densitometry of NLRP3 (**d**), ***p = 0.0001, ***p = 0.0001, and mature IL-1β (**e**), ***p = 0.0001, ***p = 0.0001 protein levels normalized to β-actin levels; *n* = 3 blots per group, each with independent samples. **f** IL-1β protein levels in the ON analyzed by ELISA, **p = 0.0092; *n* = 8 samples per group. **g** Western blot analysis of ARRB2 protein levels in the retina. **h** Quantification by densitometry of ARRB2 protein normalized to β-actin; *n* = 3 blots per group, each with independent samples. **i** Capillary tube electrophoresis of ARRB2 protein levels in the optic nerve normalized to β-actin ***p = 0.0001, ***p = 0.0001; *n* = 3 ON per group. All bar graphs are presented as the mean ± SEM, *n* = 5–9, analyzed by two-tailed unpaired *t* test

Stimulation of HCAR by βHB ameliorates neuroinflammation via inhibition of inflammasome formation [31, 45]. To explore HCAR function in ketogenic diet-induced anti-inflammatory effects in D2 glaucoma mice, we analyzed inflammasome components NLRP3 and expression of IL-1β in keto D2 retina and ON. Consistent with inhibition of the NLRP3 inflammasome, we found significantly decreased levels of *Nlrp3* (Fig. 6a) and *Il-1β* (Fig. 6b) mRNA in keto D2 retina compared to untreated D2 retina. We measured NLRP3 and mature IL-1β protein levels by western blot, detecting significantly lower levels of NLRP3 ((Fig. 6c, d) and IL-1β (Fig. 6c, e) in keto D2 retina compared to untreated D2 retina. We also measured IL-1β protein levels in ON by ELISA and detected significantly lower levels in keto D2 ON than untreated D2 ON (Fig. 6f). HCAR1-mediated anti-inflammatory effects are dependent on its adapter protein ARRB2 that acts on the NLRP3 inflammasome and ultimately reduces inflammation [32], so we examined whether ARRB2 expression is induced in keto D2 retina and ON. We detected significantly enhanced levels of ARRB2 protein in keto retina compared to untreated D2 retina (Fig. 6g, h). We analyzed ARRB2 protein level in ON by capillary electrophoresis and found significantly increased levels of ARRB2 protein in keto ON compared to untreated D2 ON (Fig. 6i). Hence, these results demonstrate that the ketogenic diet further promotes an anti-inflammatory response by inhibiting inflammasome formation in D2 glaucoma mice.

## Discussion

Inflammation is a major pathophysiological process of secondary damage following CNS insult, including traumatic brain and spinal cord injury, Alzheimer’s disease, and Parkinson’s disease [46, 47]. Glaucoma is an age-related chronic degenerative disease with inflammatory response reminiscent of many other chronic neurodegenerative diseases. In this study, we investigated the contribution of metabolic vulnerability to the inflammation evident in D2 glaucoma mouse retina and optic nerve. Our data show that limited energy supply in the D2 ON and retina triggers the primary cellular energy sensor, AMPK, that activates NF-κB signaling and consequently induces the observed pro-inflammatory response. Treatment with ketogenic diet satisfies the energy demand, inhibits AMPK activation, and reduces inflammation. Lactate or ketones from the ketogenic diet also inhibit NLRP3 inflammasome-mediated inflammation through the HCAR1 receptor in D2 retina and ON.

Beneficial effects of the ketogenic diet have been reported in many neurodegenerative diseases including epilepsy, Alzheimer’s disease, and Parkinson’s disease [48]. Free fatty acids used in epilepsy treatment can reduce IL-1β levels, possibly through activation of PPARα [20], and the ketogenic diet metabolite βHB reduces inflammation [45]. Other than the impact of the diet on inflammation, our recent study showed the ketogenic diet directly impacts energy management by inducing mitochondrial biogenesis, reversing monocarboxylate transporter decline, and inducing a robust antioxidant response. These alterations protect RGCs and their axons from degeneration, allowing them to maintain physiological signaling to the brain [8]. These data accord with evidence that βHB treatment protects cortical neurons through maintenance of neuronal respiratory capacity [49].

We first investigated microglial activation in energy-compromised D2 retina and ON. Studies have indicated that excessive and prolonged activation of microglia leads to degeneration of neurons, including RGCs [50, 51]. We detected activated microglia as shown by morphological change, including rounded shape, increased somal size, and ramified clusters in D2 retina and ON, which is consistent with previous studies showing microglia activation in glaucomatous retina and optic nerve head [50, 52]. Next, we examined how low energy in D2 retina and ON triggers inflammation. In low energy conditions, AMPKα1 is activated by phosphorylation of its Threonine 172 residue; it then works to switch cellular processes from an anabolic to a catabolic state, thereby improving energy balance by liberating ATP [53]. Our previous study showed AMPK activation early, at 6 months in the D2 ON, when glaucoma pathology is not obvious. AMPK was more highly activated at 10 month of age, indicating sustained AMPK activation in the D2 ON [8]. Activated AMPK contributes to mechanisms of inflammation [53]. Knockdown of AMPKα1 reduced LPS-induced NF-κB activation and IL-1β cytokine release [22, 23]. NF-κB is a master transcription factor for the production of cytokines; when activated, NF-κB translocates to the nucleus and induces the transcription of multiple inflammatory cytokines [54, 55]. We observed reduced NF-κB p65 activation and reduced release of pro-inflammatory cytokines TNFα, IL-6, IL-1β, and NOS2 after ketogenic diet treatment. Each of these cytokines has been associated with glaucoma pathogenesis in other studies; for example, TNFα is released by retinal glia and leads to RGC axon degeneration [56] through the upregulation of Ca^++^-permeable AMPA receptors [57]. IL-6 is synthesized by RGCs following elevation of intraocular pressure [58]. IL-1β is upregulated prior to axon degeneration or RGC loss in the D2 model of glaucoma [52]. Moreover, we observed increased expression of the anti-inflammatory cytokines IL-4 and Arginase-1 in keto D2 retina and ON. Therefore, our data suggests that the ketogenic diet-induced anti-inflammatory response is due to inhibition of AMPK-mediated NF-κB signaling in D2 glaucoma mice. Our data are consistent with a recent study showing ketogenic diet-mediated inhibition of NF-κB signaling and attenuation of inflammation after spinal cord injury [18].

Inflammation has many feed-forward mechanisms for signal amplification, many of which involve cytokine binding to toll-like receptors (TLRs) [59] that promote NF-κB translocation and greater production of cytokines. High levels of TLR expression were reported in glaucomatous human retina as well as in the D2 retina and optic nerve head [60, 61]. Given this, we investigated a pathway downstream of TLR signaling and NF-κB, the NLRP3 inflammasome. An activating signal from a pattern recognition receptor (PRP) on the cell surface leads to the upregulation of NLRP3 as a priming reaction. This activating signal can originate from many different stimuli, including HCAR1 (see below). In fact, mechanical strain of astrocytes, as might occur in optic nerve head glial cells in glaucoma, can trigger the NLRP3 inflammasome [62]. Activated TLRs stimulate a protein cascade that results in NLRP3 inflammasome assembly, which activates caspase-1, the enzyme responsible for the maturation and release of pro-inflammatory cytokine IL-1β [32, 33]. The reduction in NF-κB signaling in our data indicates that pro-form IL-1β should be reduced, but any existing pools of IL-1β could become mature through NLRP3 inflammasome activity. Interestingly, in the context of type 2 diabetes, the NLRP3 inflammasome is a sensor of metabolic stress [63]. We observed downregulation of the NLRP3 inflammasome with the ketogenic diet. Our data also shows that NLRP3 inflammasome inhibition may occur through the activity of lactate and ketone bodies on HCAR1.

Lactate or ketone body binding to the HCARs on macrophages or monocytes can also reduce inflammation [32, 45]. We detected significantly increased mRNA and protein levels of HCAR1 but not HCAR2 in D2 retina and ON, indicating that ketogenic diet treatment induces HCAR1 expression in glaucoma mice. The HCAR1 expression was observed throughout the retina. Higher levels of HCAR1 have been detected at excitatory synapses than vascular endothelium and astrocytic end feet in the retina, suggesting that HCAR1 signaling is important for various cellular functions including metabolism, synaptic function, and blood flow in the retina [29]. Lactate or ketone body-induced anti-inflammatory effects through HCAR1 require the cytosolic signaling molecule ARRB2 [64]. We observed significantly increased ARRB2 protein levels in both the keto retina and ON, demonstrating that ARRB2 expression is induced by the ketogenic diet. ARRB2 directly interacts with HCAR1 and exerts an anti-inflammatory effect by antagonizing NLRP3 inflammasome pathways [65]. A co-immunoprecipitation study suggested that ARRB2 interacts directly with NLRP3; accordingly, knockdown of ARRB2 markedly diminishes its ability to inhibit NLRP3 [66]. We observed reduced expression of NLRP3 after the ketogenic diet treatment in D2 retina and ON, which suggests lactate or ketone bodies trigger HCAR1-ARRB2 signaling to reduce the expression of the NLRP3 inflammasome. Our findings are consistent with the study showing HCAR1-mediated inhibition of the NLRP3 inflammasome in liver and pancreatic injury [31]. βHB-induced inhibition of NLRP3 inflammasome in macrophages was shown to be independent of HCAR2 signaling [45], but the study did not rule out the possibility that HCAR1 could be involved, as our data suggests. We detected significantly reduced levels of mature Il-1β after ketogenic diet treatment that corroborates HCAR1-mediated inhibition of NLRP3 inflammasome in D2 glaucoma mice. Knockdown of HCAR1 strongly induces hepatic IL-1β and NLRP3 expression and increases hepatocyte apoptosis, indicating a critical role for HCAR1 in endogenous dampening of inflammatory response [31]. Therefore, our data suggests that the ketogenic diet exerts its anti-inflammatory response in glaucoma not only through the inhibition of AMPK-NF-κB signaling but also via induction of HCAR1-mediated inhibition of NLRP3 inflammasome.

## Conclusion

These data provide evidence that metabolic vulnerability observed in glaucoma contributes to an extended inflammatory response through chronic stimulation of AMPK that activates NF-κB signaling and subsequently induces pro-inflammatory response. Treatment with the ketogenic diet resolves energy demand and ameliorates the inflammation by inhibition of AMPK activation and by activating HCAR1 signaling that inhibits NLRP3 inflammasome-mediated inflammation. Hence, these findings reveal a neuroprotective mechanism of ketogenic diet in regulating inflammation in chronic glaucoma and provide potential therapeutic targets of glaucoma and other related inflammatory diseases.

## Abbreviations

AMPK: AMP-activated protein kinase
ARRB2: Arrestin β-2
cAMP: Cyclic adenosine monophosphate
D2: DBA/2J mouse strain
D2G: DBA/2J-*Gpnmb* + mouse strain
ELISA: Enzyme-linked immunosorbent assay
GPR109A: G-protein-coupled receptor 109 A
GPR109B: G-protein-coupled receptor 109 B
GPR81: G-protein-coupled receptor 81
HCAR: Hydroxycarboxylic acid receptor
IL-1β: Interlekin-1 beta
IL-4: Interlukin-4
IL-6: Interlukin-6
IOP: Intraocular pressure
IκB: I kappa B
keto: Ketogenic diet
NF-κB: Nuclear factor-kappa B
NLRP3: NLR family, pyrin domain containing 3
NMNAT2: Nicotinamide mononucleotide adenylyltransferase 2
NOS2: Nitric oxide synthase 2
Nrf2: Nuclear factor (erythroid-derived 2)-like 2
ON: Optic nerve
PPARα: Peroxisome proliferator-activated receptor alpha
qRT-PCR: Quantitative real-time polymerase chain reaction
RGC: Retinal ganglion cell
ROI: Region of interest
TLRs: Toll-like receptors
TNF-α: Tumor necrosis factor alpha
Unt: Untreated
βHB: β-Hydroxybutyrate
